# A striking new species of *Dolichomitus* Smith, 1877 (Hymenoptera: Ichneumonidae; Pimplinae) from South America

**DOI:** 10.3897/BDJ.9.e67438

**Published:** 2021-05-28

**Authors:** Filippo Di Giovanni, Diego Galvão Pádua, Rodrigo Oliveira Araujo, Alvaro Doria Santos, Ilari Eerikki Sääksjärvi

**Affiliations:** 1 Dept. of Agriculture, Food and Environment, University of Pisa, Pisa, Italy Dept. of Agriculture, Food and Environment, University of Pisa Pisa Italy; 2 Programa de Pós-Graduaçao em Entomologia, Instituto Nacional de Pesquisas da Amazônia, Manaus, Brazil Programa de Pós-Graduaçao em Entomologia, Instituto Nacional de Pesquisas da Amazônia Manaus Brazil; 3 Centro de Investigación de Estudios Avanzados del Maule, Vicerrectoría de Investigación y Postgrado, Universidad Católica del Maule, Talca, Chile Centro de Investigación de Estudios Avanzados del Maule, Vicerrectoría de Investigación y Postgrado, Universidad Católica del Maule Talca Chile; 4 Museu de Zoologia, Universidade de São Paulo, São Paulo, Brazil Museu de Zoologia, Universidade de São Paulo São Paulo Brazil; 5 Biodiversity Unit, University of Turku, Turku, Finland Biodiversity Unit, University of Turku Turku Finland

**Keywords:** Darwin wasps, Ephialtini, idiobiont, iridescence, parasitoid, cloud forests, rain forest, Amazonia, Andes, Neotropics, tropical

## Abstract

**Background:**

*Dolichomitus* Smith, 1877 is a genus of Darwin wasps characterised by their large bodies and long ovipositors, that includes more than 75 species worldwide, 20 of which occur in the Neotropical Region. Due to recent efforts, the number of species of this genus continues to increase in South America.

**New information:**

A new iridescent species of Pimplinae, *Dolichomitus meii* sp. nov., is described and illustrated. It was discovered from the biological station of Yanayacu located in the north-eastern slopes of the tropical Andes. It may be separated from all other species of *Dolichomitus* Smith by the striking colour pattern and apically strongly decurved ovipositor.

## Introduction

*Dolichomitus* Smith is a widespread genus of pimpline Darwin wasps, represented in the Neotropical Region by 20 described species (Yu et al. 2016, Araujo et al. 2020). The tropical species of the genus are moderately large (fore wing length from 5.0 to 22.0 mm), vividly coloured and possess long ovipositors (Gauld 1991, da Silva Loffredo and Penteado-Dias 2012, Araujo et al. 2020). These characteristics make them especially conspicuous amongst the tropical Darwin wasps.

Structurally, *Dolichomitus* rather closely resembles *Anastelgis* Townes and *Umanella* Gauld in South America. All three genera contain moderately large species with long ovipositors and strong oblique grooves cutting off triangular areas on the anterolateral corners of the second metasomal tergite. *Dolichomitus* may be easily separated from *Anastelgis* by the set of the following characters: 1) apex of upper ovipositor valve simple, without tiny denticles (with a row of small denticles in *Anastelgis*), 2) lower valve of ovipositor laterally expanded to partially enclose the upper valve (lower valve not expanded in *Anastelgis*) and 3) mid-coxae of males often with prominences which are separated by deep concavities (coxae simple in *Anastelgis*). *Umanella*, on the other hand, with only three known species (Gauld 1991, Broad et al. 2010, Herrera-Florez and Molina 2018), is a very distinctive metallic blue pimpline genus which is also characterised by, for example, a very long first metasomal segment.

The Central American species of *Dolichomitus* may be identified with the key provided by Gauld (1991) and Gauld et al. (1998). The South American species may be identified using the key provided by Araujo et al. (2020) and the current article.

The aim of the present study is to describe a striking new species of *Dolichomitus* from the tropical Ecuadorian Andes. In our opinion, the species is amongst the most spectacular tropical *Dolichomitus* ever described.

## Materials and methods

Morphological terminology follows Broad et al. (2018). The information contained in “Type Material” section corresponds to the specimen label *verbatim*. The holotype of the new species is deposited in the Museum of Entomology of “Sapienza”, University of Rome, Italy (MZUR) and the paratype in Zoological Museum, Biodiversity Unit, University of Turku, Finland (ZMUT). The holotype was collected as part of a larger project carried out by "Sapienza", University of Rome (Italy) on the study of insect biodiversity in Ecuador (collecting permit n° MAE-DNB-CM-2016-0045, export authorisation n° 097-17-EXP-IC-FAU-DNB/MA). High-resolution photographs were taken at different focal stages with a Nikon D5300 digital camera attached to a Leica Z16 APO stereoscope. Images were acquired using StackShoot TM multiple-focus imaging system and stacked in a single in-focus image using Zerene Stacker software (version 1.04). Morphological measurements of the species were made with a Leica Z16 APO stereoscope containing a reticle micrometer ruler. Minor corrections at the images were made using the software Adobe Photoshop 2020.

## Taxon treatments

### Dolichomitus meii

Di Giovanni & Sääksjärvi sp. n.

EBFC7B6B-88C1-569D-AC40-5756DA43D7DB

0039a730-e645-4486-bbb3-347477cb27c6

#### Materials

- **Type status:** Holotype. **Occurrence:** individualCount: 1; sex: female; lifeStage: adult; **Location:** locationID: Napo, Cosanga, Estación Cientifica Yanayacu; higherGeography: South America; country: Ecuador; verbatimElevation: 2154 m a.s.l.; verbatimCoordinates: 0 35 25.55S 77 52 58.59W; **Event:** eventID: MAE-DNB-ARGG-CM-2016-045-001; samplingProtocol: sweep net; samplingEffort: P. Cerretti & M. Mei leg.; year: 2017; month: 12; day: 18-19; **Record Level:** institutionCode: MZUR
- **Type status:** Paratype. **Occurrence:** individualCount: 1; sex: female; lifeStage: adult; **Location:** locationID: Napo, Est. Biol. Yanayacu; higherGeography: South America; country: Ecuador; verbatimElevation: 2000 m a.s.l.; verbatimCoordinates: 0 34 20S 77 52 20W; **Event:** samplingEffort: C. Castillo leg.; year: 2006; month: 11; day: 21-2; **Record Level:** institutionCode: ZMUT

#### Description

**Figure 1.**
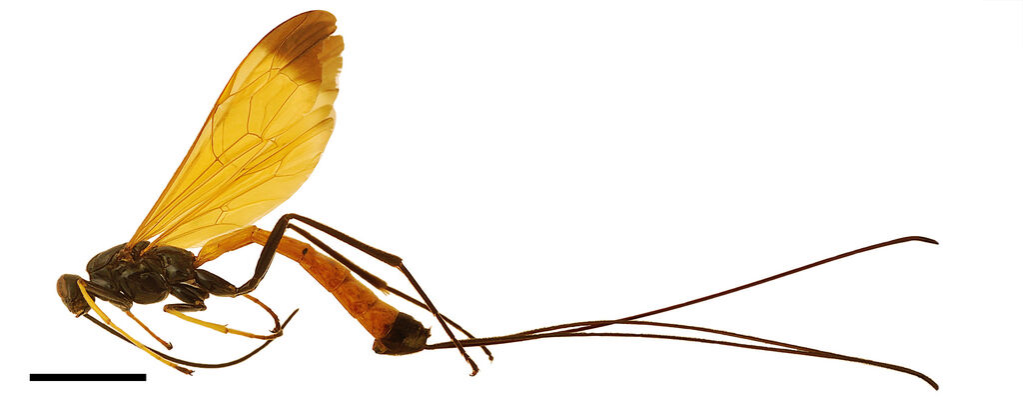
*Dolichomitus meii* sp. nov. (holotype, female). Habitus in lateral view. Scale bar: 5.00 mm.

Holotype, female (Fig. 1). Body length (without the ovipositor) about 22.0 mm; ovipositor length about 24.0 mm; fore wing length about 17.0 mm. Body covered with short white pubescence.

**Head**. Face about 0.7 times as high as wide, smooth and shiny, with small and setiferous punctures, distance between punctures greater than their diameter; compound eye slightly convergent ventrally (Fig. 2a). Frons smooth and shiny, impunctate; vertex and gena smooth and shiny, with small and setiferous punctures, distance between punctures greater than their diameter; in lateral view, gena about 0.4× as long as transverse diameter of compound eye; in dorsal view, temple linearly narrowed behind eye, about 0.60× as long as eye; distance between posterior ocellus and eye about 1.30× as long as its maximum diameter; interocellar distance about 0.90× the maximum diameter of posterior ocellus (Fig. 2b). Clypeus separated from face by a groove, smooth and impunctate, except for just a few isolated setiferous punctures along its anterior margin; clypeus about 3.50× as broad as medially high; posterior margin of clypeus impressed and bilobed, deeply excised in the middle. Malar space about 0.70× as long as basal width of mandible. Mandible with coriaceous microsculpture at the base and with setiferous punctures, smooth near teeth; mandible teeth equal. Occipital carina complete, slightly dipped dorsally, joining hypostomal carina clearly before mandible base. Antenna with 38 flagellomeres, first flagellomere about 3.60× as long as distally wide and about 1.50× as long as the second flagellomere.

**Mesosoma**. Pronotum smooth and shiny, impunctate; epomia small (Fig. 2c). Mesoscutum smooth and shiny, with small setiferous punctures, distance between punctures greater than their diameter; notauli deeply impressed at anterior 0.33 of mesoscutum, dividing it into three distinct lobes; scutellum and postscutellum smooth and shiny, with small setiferous punctures, scutellum without lateral carinae. Mesopleuron smooth and shiny, upper anterior half with small and shallow punctures, distance between punctures greater than their diameter, upper posterior half impunctate; lower half of mesopleuron with small and shallow punctures, punctures scattered, distance between them more than three times their diameter; epicnemial carina present, but very thin, not reaching the anterior margin of mesopleuron; posterior transverse carina of mesosternum absent. Metapleuron smooth and shiny, with scattered small and shallow punctures on upper half, almost impunctate on lower half; submetapleural carina complete, reaching 0.75× metapleuron length, not produced into an evident lobe anteriorly, its anterior end curved up and reaching 0.2× metapleuron height. Propodeum smooth and shiny, in dorsal view about 1.10× as long as medially wide, with small and shallow punctures on basal 0.25, apical 0.75 impunctate; propodeal spiracle elongated; propodeal carinae absent, except for pleural carina thin. Fore wing with areolet trapezoidal, vein 2rs-m about 1.10× as long as 3rs-m, cu-a opposite M&Rs (Fig. 2d); length of CU between 1m-cu&M and 2cu-a about 2.60× as long as 2cu-a. Hind wing with distal abscissa of CU present; length of proximal abscissa of CU about 0.30× as long as cu-a, proximal abscissa of CU vertical, cu-a reclivous and straight. Hind coxa smooth and polished, almost impunctate dorsally and with few small, scattered and setiferous punctures ventrally; hind femur about 5.20× as long as its maximum width.

**Metasoma**. Metasoma smooth and shiny, impunctate, only last tergites with barely discernible setiferous punctures (Fig. 2e). Tergite I about 2.20× as long as posteriorly wide, dorsolateral carina absent, but replaced by an angulation that runs from anterior to posterior margin, median longitudinal carina distinct on anterior 0.2; in lateral view, tergite I with anterior 0.33 reclivous, posterior 0.66 straight, spiracle near its anterior 0.4; tergite II about 1.60× as long as posteriorly wide, with strong oblique grooves running from anterior margin to half the tergite, that outline a clearly raised median area; tergite III about 1.10× as long as posteriorly wide, with less defined oblique grooves and a slightly raised median area; tergite IV with small tubercles laterally, near the anterior margin. Ovipositor sheath about 1.10× as long as body and 4.70× as long as hind tibia; ovipositor downcurved posteriorly, ovipositor tip with subapical dorsal lobe of lower valves not delimited posteriorly, but gradually developing into the apical teeth, dorsal lobe with an anterior groove reclivous, followed by three ridges, the first two almost vertical and the third inclivous (Fig. 2f).

**Colour**. Head, including clypeus and mandible, black, with blue reflections; palps brownish-black with last segment yellowish-brown at the proximal 0.75; antenna, scapus and pedicel black. Mesosoma, including scutellum, postscutellum, tegula and propodeum black with blue reflections. Fore wing yellowish with a distal darkened area that covers the fourth submarginal cell almost entirely; hind wing yellowish; veins and pterostigma orange. All coxae, trochanters and trochantelli black with bluish reflections, inner distal margin of all trochantelli yellowish-brown; fore femur black with bluish reflections, with a yellow line on outer surface running from proximal 0.75 to the apex; mid-femur black with blue reflections, with a yellow spot on outer side at apex; fore and mid-tibiae yellow, fore and mid-tarsi with segments yellow to gradually darker distally, last segment brown; hind femur entirely black with blue reflections, hind tibia and tarsus dull black. Metasoma with tergites I–V orange, tergite II orange with two small black spots on posterior margin just near hind corners, tergites VI+ bluish-black. Ovipositor sheath black.

#### Diagnosis

*Dolichomitus meii* sp. nov. can be distinguished from the other Neotropical species of the genus by the combination of the following characteristics: 1) body length ca. 22.0 mm, 2) bluish-black head, mesosoma and metasomal tip, with orange metasomal tergites I–V, 3) wings yellowish with distal darkened area, 4) ovipositor sheath length ca. 4.70× as long as hind tibia and 5) ovipositor tip strongly decurved, with subapical dorsal lobe of lower valves not delimited posteriorly, but gradually developing into the apical teeth, dorsal lobe with an anterior groove reclivous, followed by three ridges, the first two almost vertical and the third inclivous.

#### Etymology

The specific epithet is in honour of Maurizio Mei, a great entomologist and dear friend of FDG.

#### Distribution

Ecuador

#### Biology

Host unknown. Both type specimens have been collected approximately at the same altitude (2000 m a.s.l.).

#### Taxon discussion

*Dolichomitus meii* sp. nov. resembles *D. orejuelai* Araujo & Pádua, 2020 in colour pattern, with body partly dark and metasomal tergites I-V predominantly orange, but it differs from the latter in having mesosoma entirely black with blue reflections (mesosoma mostly reddish-black with red marks in *D. orejuelai*), metasomal tergite I and III–V entirely orange and metasomal tergite II orange with two small black spots on posterior margin just near hind corners (metasomal tergites yellowish-brown with posterior margins of tergites II–V reddish-black in *D. orejuelai*) and wings yellowish-orange with distal darkened area (wings yellowish, but without distal darkened area in *D. orejuelai*). In case of the wing colouration, *D. meii* sp. nov. keys out with *D. pimmi* Araujo & Pádua, 2020 in the updated key to the South American species of the genus, but it can be distinguished from the latter in having mesosoma entirely black with blue reflections and metasomal tergites I–V orange (mesosoma and metasoma yellow with black marks in *D. pimmi*).

#### Male

Unknown.

## Identification Keys

### Updated key to the South American species of *Dolichomitus* Smith, 1877 (adapted from Araujo et al. 2020)

**Table:** 

6	Fore wing iridescent or hyaline or yellowish, but always with apex black.	7
–	Fore wing entirely yellowish or yellowish with anterior margin more strongly yellowish.	9
7	Fore wing hyaline or iridescent.	8a
–	Fore wing yellowish.	8b
8a	Malar space 0.55× as long as proximal mandibular width; head mostly black; fore wing iridescent with strongly contrasting distal darkened area that at least covers completely the fourth submarginal cell and third discal cell, pterostigma black; metasoma mostly black shining.	***D. menai*** Araujo & Pádua
–	Malar space 0.30× as long as proximal mandibular width; head mostly yellow; fore wing hyaline with strongly contrasting distal darkened area that covers only the distal half of fourth submarginal cell, pterostigma dark brown; metasoma mostly yellowish with black lateral spots and anterior dorsal longitudinal stripe on tergite I.	***D. mariajosae*** Araujo & Pádua
8b	Mesosoma yellow with black marks; metasoma mostly orange yellow with anterior half of tergite I, posterior half of tergite V, tergites VI–VII and posterior margin of tergite VIII yellow, lateral spots and an anterior dorsal longitudinal stripe on tergite I, posterior margin of tergites I–IV, tergite VIII black and anterior margin of tergites VI and VII with a dark brown spot dorsally.	***D. pimmi*** Araujo & Pádua
–	Mesosoma black, with blue reflections; metasomal tergites I–V orange, tergite II orange with two small black spots on posterior margin just near hind corners, tergites VI+ bluish-black.	***D. meii*** Di Giovanni & Sääksjärvi, **sp. nov.**

Step 6 of the key, provided by Araujo et al. (2020), should be modified as follows:

## Discussion

Almost nothing is known about the biology of tropical American *Dolichomitus*. The ovipositor tip of *D. meii* sp. nov. is very strongly decurved; as suggested by Gauld 1991, *Dolichomitus* species may use their long ovipositors not for actual boring, but rather for threading it down pre-existing tunnels in logs. The shape of the ovipositor tip of *D. meii* sp. nov. also resembles the ovipositor tip of *Umanella*. Both genera are known to exist in Western Amazonian rainforests and tropical Andes (Herrera-Florez and Molina 2018, Araujo et al. 2020).

The conspicuous colouration of the species, with head and mesosoma shiny black with blue reflections, golden yellow wings with dark distal area and orange metasoma (with only the last tergites black), is unique. Many large-sized and metallic Darwin wasps are known to occur in South America (mainly Cryptinae and Ichneumoninae). However, within the tropical American species of the subfamily Pimplinae, the metallic blue colouration was previously only known in the genera *Pimpla* Fabricius and *Umanella*. To our knowledge, *D. meii* sp. nov. is the only metallic species of *Dolichomitus*. In case of *D. meii* sp. nov., the metallic blue colouration (reflections) of head and mesosoma is clearly visible when observing the specimens under a light source. However, it may be difficult to observe in nature.

With the description of this remarkable species, we hope to draw attention to the largely unknown, yet seemingly diverse, Darwin wasp fauna of the tropical Andes. It is obvious that our understanding of the tropical Andean Darwin wasps is incomplete. Future field studies will likely require us to re-define the current understanding of the diversity patterns of the family.

## Supplementary Material

XML Treatment for Dolichomitus meii

## Figures and Tables

**Figure 2a.**
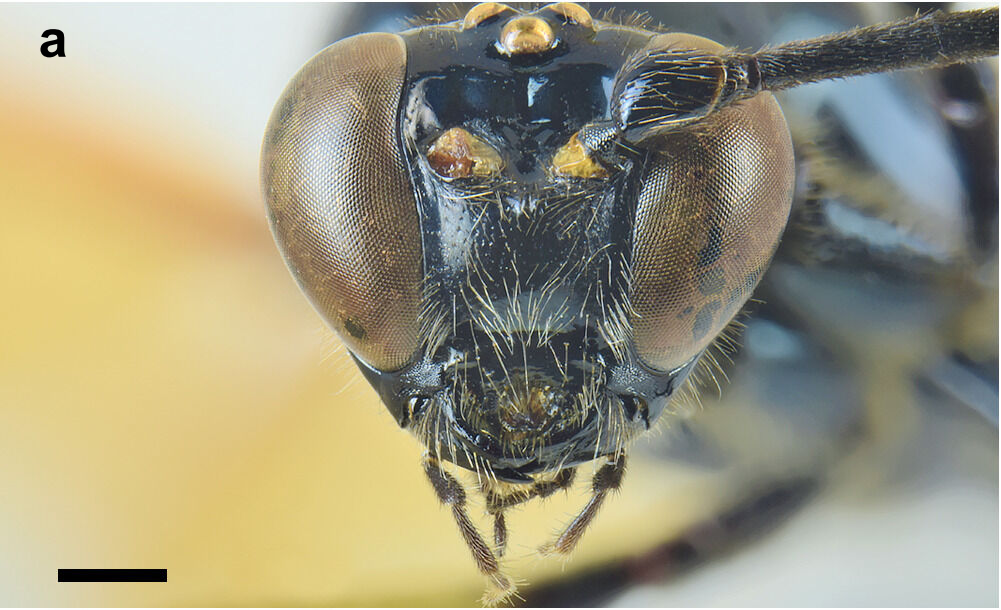
Head in frontal view

**Figure 2b.**
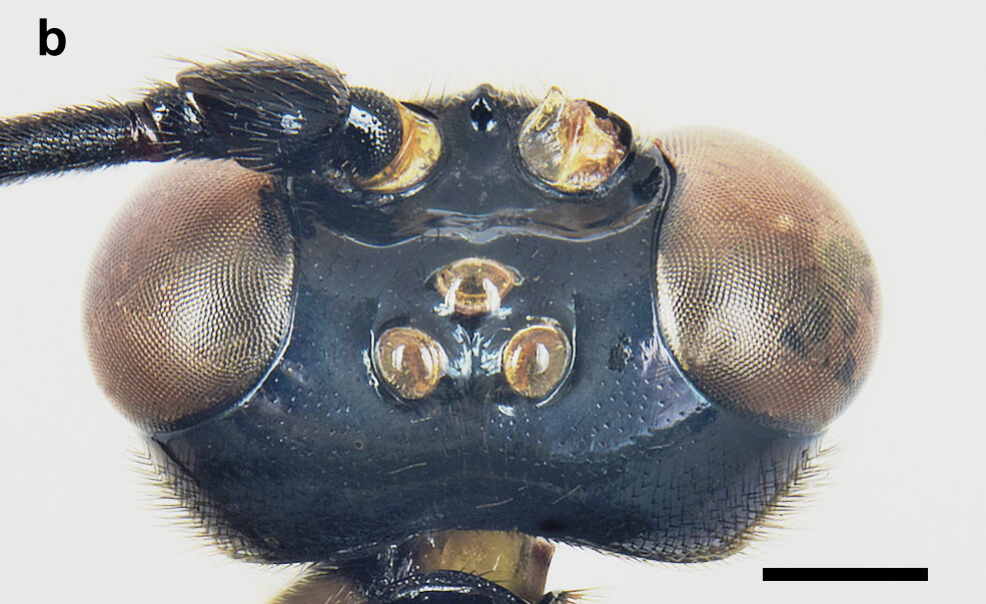
Head in dorsal view

**Figure 2c.**
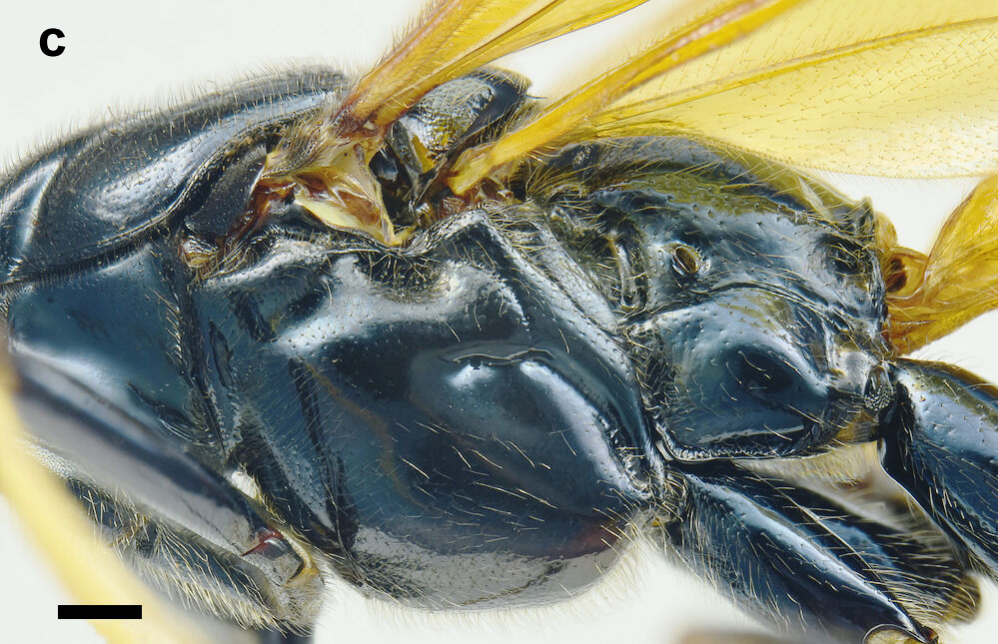
Mesosoma in lateral view

**Figure 2d.**
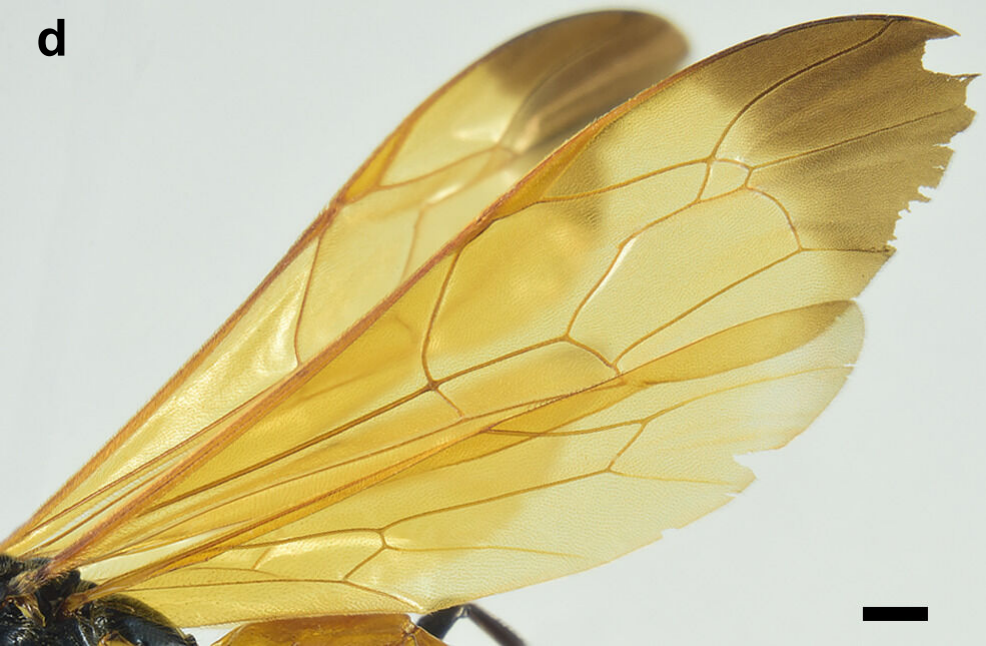
Wings

**Figure 2e.**
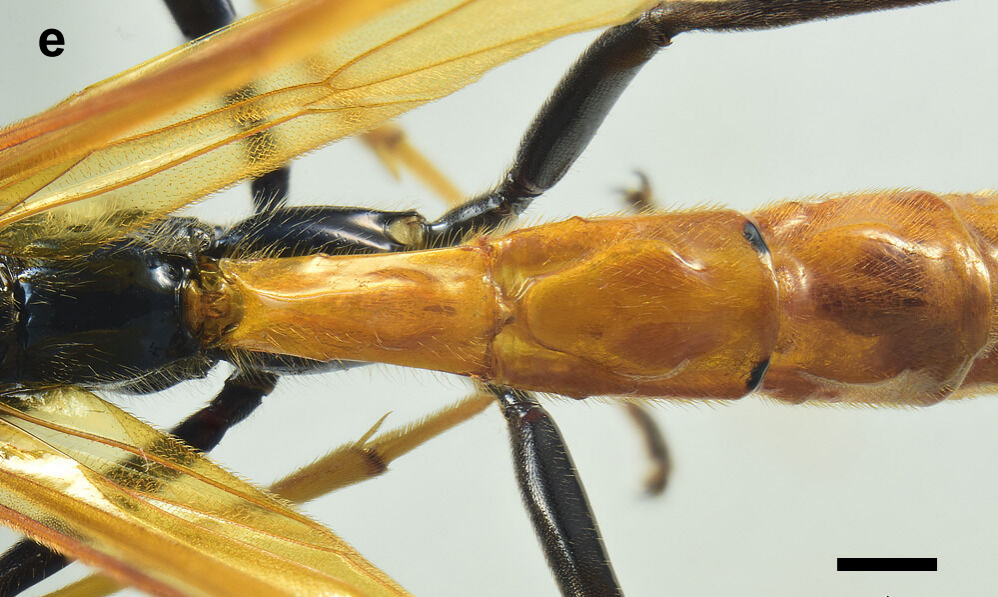
Propodeum and metasomal tergites I–III in dorsal view

**Figure 2f.**
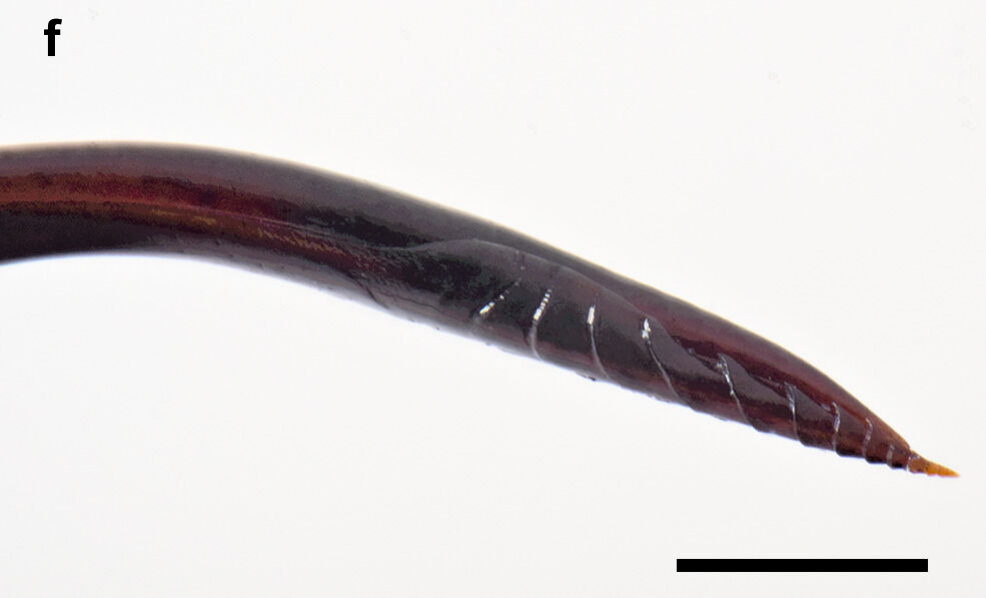
Apex of ovipositor in lateral view
